# In Vitro Activity of Eravacycline, Aztreonam–Avibactam, and Cefiderocol Against Multidrug-Resistant Gram-Negative Clinical Isolates from a Romanian Tertiary Hospital

**DOI:** 10.3390/antibiotics15100942

**Published:** 2026-09-22

**Authors:** Anda Baicus, Valentina Daniela Sisu, Andreia Domnica Tatar, Lore-Dana Barcer, Elena Andreea Birlescu, Monica Dugaesescu, Catalin Florin Cirstoiu

**Affiliations:** 1 Department of Microbiology, University of Medicine and Pharmacy, Carol Davila, 020021 Bucharest, Romania; valentina-daniela.sisu@drd.umfcd.ro (V.D.S.); andreia-domnica.tatar@drd.umfcd.ro (A.D.T.); elena-andreea.birlescu0725@rez.umfcd.ro (E.A.B.); monica.dugaesescu@drd.umfcd.ro (M.D.); 2 Clinical Laboratory, Emergency University Hospital, 050098 Bucharest, Romania; barcer.lore@suub.ro; 3 Department of Orthopedics, University of Medicine and Pharmacy, Carol Davila, 020021 Bucharest, Romania; catalin.cirstoiu@umfcd.ro; 4 Department of Orthopedics and Traumatology, Emergency University Hospital, 050098 Bucharest, Romania

**Keywords:** eravacycline, aztreonam–avibactam, cefiderocol, carbapenemase-producing Enterobacterales, *Klebsiella pneumoniae*, metallo-β-lactamase, antimicrobial susceptibility testing, antimicrobial stewardship

## Abstract

Background/Objectives: Carbapenem-resistant Gram-negative bacteria—especially NDM- and OXA-48-producing *Klebsiella pneumoniae* and carbapenem-resistant *Acinetobacter baumannii* (CRAB)—have limited treatment options, and in vitro data from Eastern Europe on the reserve agents eravacycline, aztreonam–avibactam, and cefiderocol are scarce. We evaluated their in vitro activity against consecutive multidrug-resistant (MDR) Gram-negative isolates from a Romanian tertiary hospital, stratified by species and carbapenemase profile. Methods: Seventy-seven consecutive, non-duplicate MDR Gram-negative isolates were collected over a 12-week period (January–March 2026) at a single tertiary center and tested by gradient diffusion (Liofilchem MIC Test Strip). MTS was used as an exploratory screening method; reference broth microdilution (BMD) remains the standard for categorical susceptibility confirmation. MICs were interpreted using FDA and EUCAST v16.0 breakpoints. Carbapenemases were detected by immunochromatography (18 isolates), and carbapenemase-encoding genes were detected by Xpert Carba-R PCR (40 isolates of *K. pneumoniae*). Susceptibility is reported with Wilson 95% confidence intervals. Results: *K. pneumoniae* (*n* = 51) predominated; NDM+OXA-48 was the most common profile (43.1%), and NDM was present in 64.7%. Eravacycline inhibited 90.2% of *K. pneumoniae*, and aztreonam–avibactam inhibited 92.2%, both covering all NDM-only isolates (9/9). Cefiderocol inhibited 84.3% of *K. pneumoniae* overall, but only 6/9 (66.7%) of NDM-only producers versus 20/22 (90.9%) of NDM+OXA-48 co-producers, whereas eravacycline and aztreonam–avibactam remained active against all nine NDM-only isolates. Cefiderocol was the only agent active against *P. aeruginosa* (91.7%); aztreonam–avibactam showed uniformly high MICs against non-fermenters, and all 10 CRAB isolates had low eravacycline MICs (MIC50 0.064 µg/mL). Conclusions: The three agents were complementary: aztreonam–avibactam and eravacycline covered NDM-producing *K. pneumoniae*, whereas cefiderocol was less active in vitro; cefiderocol alone covered *P. aeruginosa*. Because MICs were generated by gradient diffusion, these exploratory benchmarks require confirmation by reference broth microdilution; gradient strips agree well with BMD for aztreonam–avibactam but show weaker, agent-dependent agreement for eravacycline and cefiderocol, so they are not yet a validated substitute across all three agents.

## 1. Introduction

The World Health Organization (WHO) and the U.S. Centers for Disease Control and Prevention consider antimicrobial resistance in Gram-negative bacteria a critical threat [1,2]. Carbapenem-resistant Enterobacterales (CRE), *Acinetobacter baumannii* (CRAB), and *Pseudomonas aeruginosa* (CRPA) are associated with high mortality and limited treatment options [3]. β-lactam–β-lactamase inhibitor combinations such as meropenem–vaborbactam, imipenem–relebactam, and ceftazidime–avibactam are active against serine carbapenemases produced by CRE and *P. aeruginosa*, but none inhibit metallo-β-lactamases (MBLs) or the carbapenemases of *A. baumannii*. Newer reserve agents with distinct mechanisms have therefore been developed, including the fluorocycline eravacycline, the monobactam/β-lactamase-inhibitor combination aztreonam–avibactam, and the siderophore cephalosporin cefiderocol.

The rapid spread of OXA-48- and NDM-producing Enterobacterales has been documented in Romania and across Eastern Europe [4,5]. NDM hydrolyzes most β-lactams, including carbapenems, but not the monobactam aztreonam; OXA-48 has a narrower spectrum. Their co-occurrence on mobile genetic elements in *K. pneumoniae* yields isolates resistant to nearly all β-lactam–β-lactamase-inhibitor combinations. *A. baumannii* infections in Romanian hospitals are predominantly caused by OXA-23- and OXA-40/58-producing clones, often co-producing NDM, resulting in extensively drug-resistant phenotypes for which colistin and sulbactam-based regimens have been last-resort options [6,7].

Eravacycline (Xerava) is a fully synthetic fluorocycline approved by the FDA and the EMA in 2018 for complicated intra-abdominal infections; it retains in vitro activity against several MDR Gram-negative organisms, including CRAB, although clinical breakpoints and outcome data for CRAB remain limited [8,9]. It overcomes tetracycline-specific efflux pumps and ribosomal protection, and its MICs are typically two- to four-fold lower than those of tigecycline against CRAB.

Aztreonam–avibactam (Emblaveo) combines aztreonam—a monobactam intrinsically stable to MBLs—with avibactam, a non-β-lactam inhibitor of Ambler class A and C and selected class D (including OXA-48-like) β-lactamases [10,11]. This pairing is particularly relevant for NDM+OXA-48 co-producers: avibactam shields aztreonam from serine β-lactamase-mediated hydrolysis, while aztreonam resists NDM, making the combination effective where other β-lactams fail [12]. The EMA approved it in April 2024 for complicated intra-abdominal infections, hospital-acquired and ventilator-associated pneumonia, complicated urinary tract infections, and infections due to aerobic Gram-negative organisms with limited treatment options; the FDA approval (February 2025) is narrower, covering complicated intra-abdominal infections—used in combination with metronidazole for adults with limited or no alternative treatment options [13,14].

Cefiderocol (Fetroja) is a siderophore cephalosporin that uses bacterial iron-transport systems to cross the outer membrane and is stable against serine and metallo-carbapenemases. It is approved for complicated urinary tract infections and hospital-acquired/ventilator-associated pneumonia caused by Gram-negative organisms with limited treatment options. However, its activity is unevenly affected by NDM production and by mutations in iron-uptake systems, so susceptibility must be established by validated methods.

We previously characterized the antimicrobial susceptibility and molecular epidemiology of carbapenemase-producing *K. pneumoniae* at the same Romanian tertiary hospital, where OXA-48 (73.8%) and co-production of OXA-48 and NDM (13.1%) predominated [4], and evaluated cefiderocol against MDR Gram-negative isolates during 2024–2025 [15]. The present study extends local surveillance to a new patient cohort in 2026 and, for the first time in this setting, compares the three most recently approved reserve agents head-to-head using the same consecutive isolates.

The aim was to evaluate the in vitro activity of eravacycline, aztreonam–avibactam, and cefiderocol against consecutive MDR Gram-negative clinical isolates from a Romanian tertiary hospital, with susceptibility stratified by species and carbapenemase profile.

## 2. Results

### 2.1. Isolate Characteristics

Seventy-seven consecutive, non-duplicate Gram-negative MDR isolates were included: *K. pneumoniae* (*n* = 51, 66.2%), *P. aeruginosa* (*n* = 12, 15.6%), *A. baumannii* (*n* = 10, 13.0%), and four species represented by a single isolate each (*E. coli*, *E. cloacae*, *P. mirabilis, Providencia stuartii*). The intensive care unit (ICU) was the leading source (*n* = 32, 41.6%), followed by internal medicine (*n* = 6), general surgery (*n* = 6), nephrology (*n* = 5), neurology (*n* = 5), orthopedics (*n* = 4), gastroenterology (*n* = 4), and other wards (*n* = 15, each contributing ≤2 isolates). Urine was the most common specimen (*n* = 49, 63.6%), followed by tracheal secretions (*n* = 11, 14.3%), bronchoscopy samples (*n* = 4, 5.2%), and other specimens (*n* = 13, 16.9%). All 77 isolates were tested for the three study agents by gradient diffusion. Carbapenemase testing followed the routine laboratory algorithm rather than a study-specific protocol: 18 isolates were tested by lateral-flow immunochromatography, and 40 of the 51 *K. pneumoniae* isolates were tested by Xpert Carba-R PCR. The six *K. pneumoniae* isolates that were not characterized had not been referred for confirmatory carbapenemase testing during routine care.

### 2.2. Carbapenemase Distribution

Among *K. pneumoniae* (*n* = 51), carbapenemases or carbapenemase genes were detected in 41 (80.4%). The predominant profile was NDM+OXA-48 (*n* = 22, 43.1%), followed by NDM alone (*n* = 9, 17.6%), OXA-48 alone (*n* = 7, 13.7%), and NDM+VIM, NDM+KPC, and VIM+OXA-48 (*n* = 1 each). Four isolates (7.8%) tested negative for all targets, and six (11.8%) were not characterized. NDM was thus present in 33 of 51 *K. pneumoniae* (64.7%), alone or in combination. Among *A. baumannii* (*n* = 10), OXA-23 was identified in four isolates, OXA-40/58 in two, OXA-23+OXA-40/58 in two, and NDM+OXA-23 in two. The six uncharacterized isolates were *K. pneumoniae* for which neither immunochromatography nor Xpert Carba-R had been performed, because they had not been referred for confirmatory carbapenemase testing during routine care.

### 2.3. In Vitro Activity of Eravacycline

Eravacycline inhibited 90.2% of *K. pneumoniae* (46/51; 95% CI 79.0–95.7; MIC_50_/MIC_90_ 0.19/0.50 µg/mL; Table 1). Five isolates (9.8%) were not susceptible (MICs 1, 3, 4, 4, and >32 µg/mL). By carbapenemase profile (Table 2), susceptibility was high across NDM-containing profiles: 9/9 NDM-only (100%; 95% CI 70.1–100; MIC range 0.025–0.50 µg/mL), 21/22 NDM+OXA-48 (95.5%; 95% CI 78.2–99.2), and 1/1 each for NDM+VIM and NDM+KPC. Among OXA-48-only producers, 6/7 were susceptible; the single non-susceptible isolate had an MIC > 32 µg/mL, and its mechanism was not investigated. The VIM+OXA-48 isolate was not susceptible (MIC 3 µg/mL). Of the five non-susceptible *K. pneumoniae*, three carried OXA-type enzymes (OXA-48, NDM+OXA-48, VIM+OXA-48) and two were not characterized.

For *A. baumannii* (*n* = 10), MICs were uniformly low (range 0.032–0.47 µg/mL; MIC_50_ 0.064, MIC_90_ 0.125 µg/mL), with no isolate exceeding 0.5 µg/mL across all carbapenemase profiles, including NDM+OXA-23 co-producers—although no clinical breakpoint permits categorical interpretation. For *P. aeruginosa* (*n* = 12), MICs ranged from 1.5–>32 µg/mL (MIC_50_ 8 µg/mL), reflecting intrinsic non-susceptibility.

### 2.4. In Vitro Activity of Aztreonam–Avibactam

Aztreonam–avibactam inhibited 92.2% of *K. pneumoniae* isolates (47/51; 95% CI 81.5–96.9; MIC_50_/MIC_90_ 0.25/0.94 µg/mL). Four isolates (7.8%) were resistant (MIC > 4/4 µg/mL: 16, 24, 24, and >256 µg/mL). All nine NDM-only isolates were susceptible (100%; 95% CI 70.1–100; MIC range 0.032–0.38 µg/mL), and 21/22 NDM+OXA-48 isolates (95.5%); the single resistant NDM+OXA-48 isolate had an MIC of 16 µg/mL. NDM+VIM and NDM+KPC isolates were susceptible. Among OXA-48-only producers, 6/7 were susceptible; the resistant isolate had an MIC > 256 µg/mL, suggesting additional, uninvestigated resistance mechanisms.

The VIM+OXA-48 *K. pneumoniae* strain was resistant (MIC 24 µg/mL). A VIM+OXA-48 strain should, in principle, be susceptible—avibactam inhibits OXA-48 and aztreonam resists VIM—so an MIC of 24 µg/mL suggests additional mechanisms, such as porin loss, PBP3 modification, or coexisting enzymes that hydrolyze aztreonam despite avibactam. Carbapenemase-negative *K. pneumoniae* were uniformly susceptible (4/4). *A. baumannii* and *P. aeruginosa* had uniformly high aztreonam–avibactam MICs (MIC_50_ > 256 and 32 µg/mL, respectively), consistent with the known lack of activity against non-fermenters; no EUCAST breakpoint permits categorical interpretation for these species.

### 2.5. In Vitro Activity of Cefiderocol

Cefiderocol inhibited 84.3% of *K. pneumoniae* (43/51; 95% CI 72.0–91.8; MIC_50_/MIC_90_ 0.38/4 µg/mL); eight isolates (15.7%) were resistant. Activity varied by carbapenemase profile in a pattern that differed from the other two agents (Table 2). Against NDM-only producers, cefiderocol susceptibility was 6/9 (66.7%; 95% CI 35.4–87.9)—the same subgroup in which all isolates were susceptible to eravacycline and aztreonam–avibactam—although this estimate is based on only nine isolates and its wide confidence interval precludes generalization; 20/22 NDM+OXA-48 isolates were susceptible (90.9%; 95% CI 72.2–97.5). Among the 33 NDM-containing *K. pneumoniae*, cefiderocol inhibited 84.8% (28/33) versus 97.0% (32/33) for both eravacycline and aztreonam–avibactam.

Cefiderocol was the only agent with meaningful activity against *P. aeruginosa*, inhibiting 11/12 isolates (91.7%; 95% CI 64.6–98.5; MIC50 0.25 µg/mL); the single non-susceptible isolate had an MIC of 4 µg/mL. Against *A. baumannii*, MICs were heterogeneous (range 0.094–>256 µg/mL; MIC_50_ 1.5 µg/mL), with two isolates > 256 µg/mL; the absence of an EUCAST breakpoint precludes categorical interpretation. Of the eight cefiderocol-resistant *K. pneumoniae* isolates, five carried NDM (with or without OXA-48)—consistent with the recognized effect of MBL production and compatible with reported iron-uptake alterations, though the latter were not tested here—two were OXA-48-only producers, and one was uncharacterized. Every NDM-carrying cefiderocol-resistant isolate remained susceptible to both aztreonam–avibactam and eravacycline; the only isolate non-susceptible to all three agents was an OXA-48-only producer. With that single exception, cefiderocol resistance did not leave any *K. pneumoniae* without an active alternative agent.

Considered together, 39 of 51 *K. pneumoniae* isolates (76.5%) were susceptible to all three agents, and only one isolate—an OXA-48-only producer with eravacycline > 32, aztreonam–avibactam > 256, and cefiderocol > 256 µg/mL—was non-susceptible to all three.

## 3. Discussion

In this NDM/OXA-48-endemic Romanian collection, the three most recently approved reserve agents showed largely complementary activity. Eravacycline and aztreonam–avibactam each inhibited ~90% of *K. pneumoniae* isolates and were active against all nine NDM-only producers, whereas cefiderocol—although active overall—showed reduced activity against that NDM-only subgroup. Conversely, cefiderocol was the only agent with meaningful activity against *P. aeruginosa*, and eravacycline was the only agent with uniformly low CRAB MICs. No single agent covered the entire panel, arguing for pathogen- and mechanism-directed selection rather than a single empirical default.

### 3.1. Eravacycline Against K. pneumoniae and Enterobacterales

The 90.2% eravacycline susceptibility among *K. pneumoniae* (MIC_90_ 0.50 µg/mL at the FDA breakpoint) is consistent with multinational surveillance, in which susceptibility among MDR Enterobacterales was 84.2% [16] and MIC_90_ values of ~1 µg/mL were reported for carbapenemase-producing Enterobacterales from China and Spain [17,18,19]. Reported rates are strongly breakpoint-dependent: among NDM-producing *K. pneumoniae* from Poland, susceptibility was 100% at the ≤2 µg/mL EUCAST epidemiological cut-off (ECOFF) but 73% by the stricter FDA breakpoint [20], and Greek and Turkish series reported 61–67% [21,22]. In our collection, all nine NDM-only isolates were susceptible even at the FDA cutoff (MIC range: 0.025–0.50 µg/mL), confirming that NDM production per se does not diminish eravacycline activity—which is relevant because NDM is the dominant carbapenemase locally. The three characterized non-susceptible isolates carried OXA-type enzymes; because OXA-type carbapenemases do not hydrolyze tetracyclines, this co-occurrence is descriptive only and is not evidence of a causal link. Efflux up-regulation and ribosomal target modification are recognized causes of eravacycline non-susceptibility in Enterobacterales [23,24,25], but no efflux assays, target-site sequencing, or whole-genome sequencing (WGS) were performed, so no resistance mechanism can be assigned in these isolates.

### 3.2. Eravacycline Against A. baumannii

All 10 CRAB isolates had low eravacycline MICs (0.032–0.47 µg/mL; MIC_50_ 0.064 µg/mL), including NDM+OXA-23 and OXA-23+OXA-40/58 co-producers. This compares favorably with larger datasets (MIC_50_/MIC_90_ 0.125/0.5 µg/mL [8]; ≤0.25/1 µg/mL [19]). However, no CLSI or EUCAST breakpoints exist for eravacycline against *A. baumannii*, and a recent method comparison found only moderate strip-versus-BMD agreement for *Acinetobacter* (categorical agreement 90%, essential agreement 88%) [26]. These MICs are therefore hypothesis-generating: they support prospective evaluation of eravacycline as part of CRAB-active combination regimens—not monotherapy—and underscore the need to establish clinical breakpoints, but they cannot, by themselves, justify clinical use.

### 3.3. Aztreonam–Avibactam for NDM-Producing K. pneumoniae

A key in vitro finding is the activity of aztreonam–avibactam against NDM-producing *K. pneumoniae* (100% of NDM-only and 95% of NDM+OXA-48 isolates). Given that NDM+OXA-48 accounted for 43% of our *K. pneumoniae* isolates and NDM for 64.7% overall, these findings align well with local epidemiology and are consistent with multinational surveillance, in which essentially all MBL-positive Enterobacterales were inhibited at ≤8 µg/mL, while *P. aeruginosa* activity was poor [11,27]. Local Romanian data likewise support aztreonam-based regimens for OXA-48- and NDM-producing *K. pneumoniae* [28]. The method-comparison studies are reassuring for this agent: gradient strips showed 99.7% categorical and 90.2% essential agreement with BMD across carbapenemase genotypes [29], and a multicenter Etest evaluation reported 95.7% essential and 98.9% categorical agreement [30].

### 3.4. Cefiderocol and Its Complementarity with Aztreonam–Avibactam

Adding cefiderocol to the panel clarifies the in vitro landscape. Overall, cefiderocol’s activity against *K. pneumoniae* (84%) was lower than that of the other two agents, and the deficit was concentrated among NDM-only producers (67% susceptible), mirroring our earlier observation that NDM-only *K. pneumoniae* were resistant to cefiderocol while OXA-48 producers were largely susceptible [15]. The broader literature links MBL production and mutations in TonB-dependent siderophore receptors (e.g., cirA, fiu) that reduce drug uptake to higher cefiderocol MICs [31]. Every NDM-carrying isolate that was cefiderocol-resistant remained susceptible to aztreonam–avibactam and eravacycline, so the two agents cover cefiderocol’s principal gap. The reverse is also true for the non-fermenters: cefiderocol was the sole agent active against *P. aeruginosa* (92%), where neither eravacycline nor aztreonam–avibactam has a role. These reciprocal profiles are compatible with a mechanism-guided approach in which carbapenemase type and species may inform, rather than determine, the choice of agent. As with the other two agents, gradient-strip cefiderocol MICs must be interpreted with caution because strips and disk diffusion can misclassify isolates near the breakpoint and are affected by trailing, which tends to under-read MICs relative to iron-depleted BMD [32].

### 3.5. Mechanisms of Aztreonam–Avibactam Resistance

Two aztreonam–avibactam-resistant patterns were observed. A VIM+OXA-48 isolate had an MIC of 24 µg/mL despite a genotype that should be susceptible, implicating porin loss, PBP3 insertions, efflux, or an undetected β-lactamase; an OXA-48-only isolate had an MIC > 256 µg/mL, again suggesting a secondary mechanism beyond the inhibited enzyme. Such mechanisms—PBP3 insertions, AmpC/CMY co-production, and porin changes—are increasingly reported in NDM- and OXA-48-producing Enterobacterales and can emerge under aztreonam–avibactam exposure [31,33,34,35,36]. These interpretations are inferential, since no WGS was performed. *A. baumannii* and *P. aeruginosa* had uniformly high aztreonam–avibactam MICs, as expected. For CRAB, alternative agents with proven activity—sulbactam-durlobactam [37] and, pending breakpoints, eravacycline—warrant consideration; for CRPA, cefiderocol, ceftolozane–tazobactam or, in non-MBL producers, ceftazidime–avibactam or imipenem–relebactam are options.

### 3.6. Local Epidemiology and Stewardship Implications

The predominance of NDM+OXA-48 co-production (43%), with NDM in 64.7% of *K. pneumoniae*, reflects the endemic Eastern European clonal landscape, in which high-risk *K. pneumoniae* lineages have driven NDM/OXA-48 co-dissemination [4,38,39]. Clonal typing was not performed on the present collection, so the contribution of specific sequence types could not be assessed. Within the limits of an in vitro survey, the data are compatible with a pragmatic, mechanism-guided ordering of options for carbapenemase-producing *K. pneumoniae*: aztreonam–avibactam or eravacycline appear to be the more reliable in vitro choices for NDM-containing isolates, with cefiderocol—confirmed by a reference method—reserved for use when the other agents are unsuitable or for *P. aeruginosa*. Because the study provides no clinical outcome data and relies on a non-reference MIC method, this ordering should be read as a hypothesis for stewardship discussion and empirical-therapy review, not as a treatment recommendation or a validated hierarchy.

### 3.7. Limitations

Several limitations affect these findings. First, MICs for the three study agents were determined using gradient diffusion rather than the reference broth microdilution (BMD) method. Gradient methods can misclassify isolates within one dilution of the breakpoint, and for cefiderocol, results are further influenced by iron content and trailing. All categorical calls—particularly those near breakpoints—should be considered exploratory and verified by BMD. Method-comparison studies indicate that gradient-strip results agree closely with BMD for aztreonam–avibactam [29,30], but agreement is weaker and agent-dependent for eravacycline (notably against *A. baumannii*) and for cefiderocol [26,32,40,41]; gradient strips therefore cannot yet be recommended as a validated alternative to BMD across all three agents. Second, the sample size (*n* = 77), including subgroups such as *A. baumannii* (*n* = 10), *P. aeruginosa* (*n* = 12), and the four single-isolate species (*E. coli*, *E. cloacae*, *P. mirabilis*, *P. stuartii*), is small. Third, characterization was limited to carbapenemase families via immunochromatography and, for 40 *K. pneumoniae* isolates, by Carba-R PCR. No allelic variant typing or clonal analysis (e.g., PFGE, MLST, WGS) was conducted, so the “non-duplicate” designation cannot rule out inter-patient transmission of dominant clones, and mechanistic explanations for resistance are mostly inferential. Fourth, being a single-center study conducted over 12 weeks, predominantly involving ICU and urine isolates, limits broader applicability. Lastly, in vitro susceptibility does not confirm clinical effectiveness; PK/PD targeting and outcome data are needed before using these MICs to guide therapy.

## 4. Materials and Methods

### 4.1. Study Design and Setting

This was a single-center, cross-sectional in vitro susceptibility survey of consecutive clinical isolates conducted at the Clinical Laboratory of the Emergency University Hospital in Bucharest, Romania. Reporting follows the applicable items of the STROBE statement for cross-sectional studies. No patient-level intervention or follow-up was involved; the unit of analysis was the bacterial isolate.

### 4.2. Isolate Collection and Definition of Non-Duplicate

Consecutive MDR Gram-negative isolates recovered from clinical samples of hospitalized patients between January and March 2026 were included. In the absence of molecular clonality testing, a non-duplicate defined a non-duplicate isolate as a single isolate per patient and per species.

### 4.3. Inclusion Criteria

Isolates were included if they met at least one internationally accepted criterion: resistance to ≥1 agent across ≥3 antimicrobial categories (MDR), phenotypic carbapenem resistance, or carbapenemase production detected by immunochromatography [42]. All MDR isolates meeting these criteria during the study period were included; no additional exclusions were applied. In this cohort, all 77 isolates met the MDR definition; phenotypic carbapenem resistance and/or carbapenemase production were additional features of most isolates.

### 4.4. Identification and Routine Susceptibility Testing

Samples were inoculated onto Columbia blood agar and bromothymol lactose blue agar and incubated aerobically at 37 °C for 18–24 h. Depending on the species, identification and routine antimicrobial susceptibility testing (AST) were performed either with the BD Phoenix™ M50 automated broth-microdilution system (NMIC/ID-503 panels; Becton Dickinson, Franklin Lakes, NJ, USA) or with MALDI-TOF identification (Bruker, Daltonics, Germany) and the Sensititre™ system (Thermo Fisher Scientific, Waltham, MA, USA) using EUGNF, EUMDRXXF and EUX2NF Gram-negative panels.

### 4.5. MIC Determination for Eravacycline, Aztreonam–Avibactam, and Cefiderocol

MICs of eravacycline, aztreonam–avibactam, and cefiderocol were determined by gradient diffusion using MIC Test Strip (MTS™; Liofilchem, Roseto degli Abruzzi, Italy). Inocula were adjusted to a 0.5 McFarland standard and applied to Mueller–Hinton agar. Strips were placed within 15 min of inoculation, and plates were incubated at 35 ± 2 °C for 18–24 h. MICs were read at the intersection of the elliptical inhibition zone with the strip scale. The eravacycline strip spans 0.002–32 µg/mL; the aztreonam–avibactam strip uses a fixed avibactam concentration of 4 µg/mL with an aztreonam gradient of 0.016–256 µg/mL; the cefiderocol strip spans 0.016–256 µg/mL. Gradient diffusion was used for all three study agents. Each strip lot was quality-controlled with the reference strains *K. pneumoniae* ATCC 700603 and *P. aeruginosa* ATCC 27853, and all QC MICs fell within the CLSI/EUCAST acceptable ranges.

### 4.6. Breakpoint Interpretation

*Eravacycline/Enterobacterales*. MICs were interpreted using the FDA breakpoint: susceptible (S) ≤ 0.5 µg/mL; values > 0.5 µg/mL were classified as not susceptible (NS). The FDA defines no intermediate or resistant category, and EUCAST provides a breakpoint only for *E. coli*; accordingly, the term “resistant” is not used for eravacycline in this report. For this reason, FDA breakpoints were adopted for eravacycline, whereas EUCAST v16.0 breakpoints, which are established for both agents, were used for aztreonam–avibactam and cefiderocol.

*Eravacycline/non-fermenters.* No validated FDA, CLSI, or EUCAST clinical breakpoints exist for eravacycline against *A. baumannii*; MICs are reported without categorical interpretation. *P. aeruginosa* is intrinsically non-susceptible [43].

*Aztreonam–avibactam/Enterobacterales.* Interpreted using EUCAST v16.0 (2026) breakpoints: S ≤ 4 µg/mL, R > 4 µg/mL (avibactam fixed at 4 µg/mL); EUCAST defines no intermediate category [44]. The FDA breakpoints differ in structure (S ≤ 4/4, I 8/4, R ≥ 16/4); the two systems agree on the susceptible cut-off but not on the resistant boundary, so categorical calls are reported per EUCAST. No EUCAST breakpoint exists for aztreonam–avibactam against non-fermenters; MICs for *A. baumannii* and *P. aeruginosa* are shown for completeness.

*Cefiderocol.* Interpreted using EUCAST v16.0 (2026) breakpoints for Enterobacterales and *P. aeruginosa*: S ≤ 2 µg/mL, R > 2 µg/mL. EUCAST assigns “insufficient evidence” to cefiderocol against *A. baumannii*; MICs for that species are reported without categorical interpretation.

### 4.7. Carbapenemase Detection

Carbapenemase production was assessed for 18 isolates by lateral-flow immunochromatography: O.K.N.V.I. Resist-5 (OXA-48, KPC, NDM, VIM, IMP) for Enterobacterales and Resist Acineto (OXA-23, OXA-40, OXA-58, NDM) for non-fermenters (Coris BioConcept, Gembloux, Belgium) [45]. Forty of the 51 *K. pneumoniae* isolates were tested with the Xpert Carba-R assay (Cepheid, Sunnyvale, CA, USA), a multiplex real-time PCR that detects VIM, IMP, NDM, KPC, and OXA-48. Immunochromatography and Carba-R identify carbapenemase families but do not distinguish allelic variants (e.g., NDM-1 vs. NDM-5), detect rare MBLs such as SPM, or perform clonal typing.

### 4.8. Data Analysis

Analyses were descriptive; no formal hypothesis testing or between-group comparisons were performed, consistent with the surveillance design. Categorical variables (species, ward, specimen, carbapenemase profile) were summarized as counts and percentages. For each organism–antimicrobial combination, the full MIC distribution, MIC range, MIC_50,_ and MIC_90_ were tabulated. Interval-censored (“off-scale”) results were retained at the limiting dilution for ranking: values above the top of the gradient (>32 µg/mL for eravacycline; >256 µg/mL for aztreonam–avibactam and cefiderocol) were assigned to the highest category and treated as non-susceptible. Because MIC_90_ is unstable when few isolates are available, it is reported only for groups with ≥10 isolates; for smaller strata, only the MIC range and MIC_50_ are shown, and for single-isolate species the observed MIC is given for completeness. Susceptibility was expressed as the proportion of susceptible isolates, with 95% Wilson score CIs computed for both species-level totals and carbapenemase strata; isolates not characterized for carbapenemases were reported as a separate category rather than pooled. Calculations were performed in IBM SPSS Statistics v29.0 (IBM Corp., Armonk, NY, USA).

## 5. Conclusions

In a Romanian tertiary hospital where NDM and NDM+OXA-48 *K. pneumoniae* predominate, eravacycline and aztreonam–avibactam were active against ~90% of *K. pneumoniae*, including all nine NDM-only producers. Cefiderocol showed reduced activity in that subgroup but was the only agent active against *P. aeruginosa*. Eravacycline alone had uniformly low CRAB MICs. The three reserve agents are therefore complementary rather than interchangeable, and the findings support further evaluation of species- and mechanism-informed selection. Because these MICs were obtained by a non-reference method in a small, single-center, genotypically but not clonally characterized collection, the results are exploratory susceptibility benchmarks that require confirmation by broth microdilution and correlation with clinical outcomes before they can inform treatment.

## Figures and Tables

**Table 1:** In vitro activity of eravacycline, aztreonam–avibactam, and cefiderocol against MDR Gram-negative isolates, by species (*n* = 77).

Species	N	Eravacycline					Aztreonam–Avibactam					Cefiderocol				
		S *n* (%; 95% CI)	NS *n* (%)	MIC Range	MIC_50_	MIC_90_	S *n* (%; 95% CI)	R *n* (%)	MIC Range	MIC_50_	MIC_90_	S *n* (%; 95% CI)	R *n* (%)	MIC Range	MIC_50_	MIC_90_
*K. pneumoniae*	51	46 (90.2; 79.0–95.7)	5 (9.8)	0.025–>32	0.19	0.50	47 (92.2; 81.5–96.9)	4 (7.8)	0.032–>256	0.25	0.94	43 (84.3; 72.0–91.8)	8 (15.7)	0.016–>256	0.38	4
*P. aeruginosa*	12	– ^a^	–	1.5–>32	8	>32	– ^b^	–	12–>256	32	>256	11 (91.7; 64.6–98.5)	1 (8.3)	0.047–4	0.25	1.5
*A. baumannii*	10	– ^c^	–	0.032–0.47	0.064	0.125	– ^b^	–	16–>256	>256	>256	– ^d^	–	0.094–>256	1.5	>256
*E. coli*	1	0 (0)	1 (100)	3	3	–	1 (100)	0 (0)	0.5	0.5	–	1 (100)	0 (0)	0.125	0.125	–
*E. cloacae*	1	1 (100)	0 (0)	0.125	0.125	–	1 (100)	0 (0)	0.125	0.125	–	1 (100)	0 (0)	0.5	0.5	–
*P. mirabilis*	1	0 (0)	1 (100)	0.75	0.75	–	1 (100)	0 (0)	0.016	0.016	–	1 (100)	0 (0)	0.38	0.38	–
*P. stuartii*	1	0 (0)	1 (100)	1.5	1.5	–	0 (0)	1 (100)	>256	>256	–	0 (0)	1 (100)	3	3	–
All Enterobacterales	55	47 (85.5; 73.8–92.4)	8 (14.5)	0.025–>32	0.19	1.5	50 (90.9; 80.4–96.1)	5 (9.1)	0.016–>256	0.25	1	46 (83.6; 71.7–91.1)	9 (16.4)	0.016–>256	0.38	4

MIC values in µg/mL; gradient diffusion (Liofilchem MTS™). S, susceptible; NS, not susceptible (eravacycline); R, resistant. “S *n* (%; 95% CI)” gives the count susceptible, percentage and Wilson score 95% confidence interval (CI omitted for single-isolate species). MIC_50_/MIC_90_ = 50th/90th percentiles; MIC_90_ shown only for groups with ≥10 isolates (–otherwise). Off-scale MICs were assigned to their limiting reported value (>32 µg/mL for eravacycline; >256 µg/mL for aztreonam–avibactam and cefiderocol) and treated as non-susceptible when computing MIC_50_/MIC_90_ and percentages. ^a^ *P. aeruginosa* is intrinsically non-susceptible to eravacycline; MICs are reported without categorical interpretation. ^b^ No EUCAST aztreonam–avibactam breakpoint for *P. aeruginosa* or *A. baumannii*; MICs are reported without categorical interpretation. ^c^ No FDA or EUCAST clinical breakpoint for eravacycline against *A. baumannii*; MICs are reported without categorical interpretation. ^d^ EUCAST assigns “insufficient evidence” to cefiderocol against *A. baumannii*; MICs are reported without categorical interpretation. Breakpoints—Eravacycline (Enterobacterales): FDA S ≤ 0.5 µg/mL; >0.5 = NS (no I/R defined). Aztreonam–avibactam (Enterobacterales): EUCAST v16.0 S ≤ 4, R > 4 µg/mL. Cefiderocol (Enterobacterales*, P. aeruginosa*): EUCAST v16.0 S ≤ 2, R > 2 µg/mL.

**Table 2:** In vitro activity of eravacycline, aztreonam–avibactam and cefiderocol against *K. pneumoniae*, by carbapenemase profile (*n* = 51).

Carbapenemase	N	Eravacycline					Aztreonam–Avibactam					Cefiderocol				
		S *n* (%; 95% CI)	NS *n* (%)	MIC Range	MIC_50_	MIC_90_	S *n* (%; 95% CI)	R *n* (%)	MIC Range	MIC_50_	MIC_90_	S *n* (%; 95% CI)	R *n* (%)	MIC Range	MIC_50_	MIC_90_
NDM + OXA-48	22	21 (95.5; 78.2–99.2)	1 (4.5)	0.064–4	0.19	0.25	21 (95.5; 78.2–99.2)	1 (4.5)	0.125–16	0.25	0.50	20 (90.9; 72.2–97.5)	2 (9.1)	0.125–4	0.38	2
NDM	9	9 (100; 70.1–100)	0 (0)	0.025–0.50	0.125	–	9 (100; 70.1–100)	0 (0)	0.032–0.38	0.25	–	6 (66.7; 35.4–87.9)	3 (33.3)	0.064–8	2	–
OXA-48	7	6 (85.7; 48.7–97.4)	1 (14.3)	0.064–>32	0.19	–	6 (85.7; 48.7–97.4)	1 (14.3)	0.19–>256	0.19	–	5 (71.4; 35.9–91.8)	2 (28.6)	0.016–>256	0.75	–
NDM + VIM	1	1 (100)	0 (0)	0.38	0.38	–	1 (100)	0 (0)	0.38	0.38	–	1 (100)	0 (0)	0.38	0.38	–
NDM + KPC	1	1 (100)	0 (0)	0.094	0.094	–	1 (100)	0 (0)	0.50	0.50	–	1 (100)	0 (0)	2	2	–
VIM + OXA-48	1	0 (0)	1 (100)	3	3	–	0 (0)	1 (100)	24	24	–	1 (100)	0 (0)	0.094	0.094	–
Negative	4	4 (100; 51.0–100)	0 (0)	0.064–0.25	0.094	–	4 (100; 51.0–100)	0 (0)	0.032–0.94	0.047	–	4 (100; 51.0–100)	0 (0)	0.032–1	0.047	–
Not characterized	6	4 (66.7; 30.0–90.3)	2 (33.3)	0.19–4	0.25	–	5 (83.3; 43.6–97.0)	1 (16.7)	0.19–24	0.19	–	5 (83.3; 43.6–97.0)	1 (16.7)	0.19–>256	0.25	–
All NDM-containing	33	32 (97.0; 84.7–99.5)	1 (3.0)	0.025–4	0.19	0.25	32 (97.0; 84.7–99.5)	1 (3.0)	0.032–16	0.25	0.50	28 (84.8; 69.1–93.3)	5 (15.2)	0.064–8	0.50	3
Total *K. pneumoniae*	51	46 (90.2; 79.0–95.7)	5 (9.8)	0.025–>32	0.19	0.50	47 (92.2; 81.5–96.9)	4 (7.8)	0.032–>256	0.25	0.94	43 (84.3; 72.0–91.8)	8 (15.7)	0.016–>256	0.38	4

MIC values in µg/mL. Column structure as in Table 1. Off-scale MICs were assigned to their limiting reported value (>32 µg/mL for eravacycline; >256 µg/mL for aztreonam–avibactam and cefiderocol) and treated as non-susceptible when computing MIC_50_/MIC_90_ and percentages. For single-isolate strata, only the count/percentage of the observed isolate is shown (no CI, no MIC_90_). “Negative”: no carbapenemase detected by the method(s) performed (immunochromatography or Xpert Carba-R); “Not characterized”: neither immunochromatography nor Carba-R performed. Strata represented by a single isolate (NDM+VIM, NDM+KPC, VIM+OXA-48) are descriptive only, and no proportion, rate or confidence interval should be inferred from them. The 66.7% cefiderocol susceptibility of NDM-only producers is based on nine isolates with a wide confidence interval and is not generalizable. Breakpoints—Eravacycline (Enterobacterales): FDA S ≤ 0.5 µg/mL; >0.5 = NS (no I/R defined). Aztreonam–avibactam (Enterobacterales): EUCAST v16.0 S ≤ 4, R > 4 µg/mL. Cefiderocol (Enterobacterales): EUCAST v16.0 S ≤ 2, R > 2 µg/mL.

## Data Availability

The original contributions presented in this study are included in the article. Further inquiries can be directed to the corresponding author.
